# A Mobile Health Mindfulness Intervention for Women With Moderate to Moderately Severe Postpartum Depressive Symptoms: Feasibility Study

**DOI:** 10.2196/17405

**Published:** 2020-11-12

**Authors:** Lyndsay A Avalos, Sara Aghaee, Elaine Kurtovich, Charles Quesenberry Jr, Linda Nkemere, MegAnn K McGinnis, Ai Kubo

**Affiliations:** 1 Kaiser Permanente Northern California Division of Research Oakland, CA United States

**Keywords:** depression, postpartum, health services, mental, eHealth, mental health, internet-based intervention, mindfulness, behavioral intervention, mobile phone

## Abstract

**Background:**

Approximately 20% of women suffer from postpartum depression (PPD). Due to barriers such as limited access to care, half of the women with PPD do not receive treatment. Therefore, it is critical to identify effective and scalable interventions. Traditional mindfulness programs have been effective in reducing depressive symptoms, however access remains a barrier. A self-paced mobile health (mHealth) mindfulness program may fit the lifestyle of busy mothers who are unable to attend in-person classes. However, little is known regarding the feasibility or efficacy of mHealth mindfulness interventions in postpartum women with depressive symptoms.

**Objective:**

This study aims to assess the feasibility, acceptability, and preliminary efficacy of an mHealth mindfulness intervention for postpartum women with moderate to moderately severe depressive symptoms.

**Methods:**

We conducted a single-arm feasibility trial of an mHealth mindfulness intervention within Kaiser Permanente Northern California (KPNC), a large integrated health care system. Participants were identified through clinician referral and electronic health records via KPNC's universal perinatal depression screening program and recruited by the study team. Inclusion criteria included the following: English-speaking, up to 6 months postpartum with a Patient Health Questionnaire (PHQ-8) score of 10 to 19, and no regular mindfulness/meditation practice. Participants were asked to use a mindfulness app, Headspace, 10 to 20 min/day for 6 weeks. Baseline and postintervention surveys captured data on patient-reported outcomes (depression and stress symptoms, sleep quality, and mindfulness). Semistructured interviews captured acceptability. Retention and adherence were used to assess feasibility.

**Results:**

Of the 115 women who were contacted and met the eligibility criteria or declined participation before eligibility assessment, 27 (23%) were enrolled. In addition, 70% (19/27) completed the study. The mean age of participants was 31 years (SD 5.2), 30% (8/27) were non-Hispanic White, and, on average, participants were 12.3 weeks postpartum (SD 5.7). Of the women who completed the study, 100% (19/19) used the Headspace app at least once, and nearly half (9/19, 47%) used the app on ≥50% of the days during the 6-week intervention period. Of the 16 participants who completed the postintervention interview, 69% (11/16) reported that they were *very* or *extremely* satisfied with the app. Interviews indicated that women appreciated the variety of meditations and felt that the program led to reduced anxiety and improved sleep. Significant improvements in pre- and postintervention scores were observed for depressive symptoms (PHQ-8: −3.8, *P*=.004), perceived stress (10-item Perceived Stress Scale: −6.0, *P*=.005), and sleep quality (Pittsburgh Sleep Quality Index: −2.1, *P*=.02, indicating less sleep disturbance). Improvements in mindfulness were also significant (Five Facet Mindfulness Questionnaire-Short Form: 10.9, *P*=.01).

**Conclusions:**

An mHealth mindfulness intervention for postpartum women with moderate to moderately severe depressive symptoms is feasible and acceptable. An efficacy trial is warranted.

## Introduction

### Postpartum Depression

Postpartum depression (PPD) is the number 1 complication of childbirth [1,2], affecting up to 20% of postpartum women. It is a life-threatening, debilitating, and costly mood disorder that emerges within a year of delivery [1,3,4]. Symptoms of PPD include loss of interest or energy, depressed mood, fluctuations in sleep or eating patterns, reduced ability to think or concentrate, feelings of worthlessness, and recurrent suicidal ideation; PPD can also result in infanticide [5,6]. PPD can have multigenerational consequences, substantially affecting the health of the mother and the child. For example, women with PPD are more likely to demonstrate hostile and/or coercive behaviors and disengagement from their infants [7], resulting in negative mother-infant interactions [8]. Women with PPD are less likely to breastfeed and are at increased risk of early cessation of breastfeeding [9], and their infants receive fewer preventive services, such as recommended immunizations [10,11]. Children of women with PPD have poorer cognitive function [12-14], are at increased risk of behavioral and developmental disorders, such as attention deficit hyperactivity disorder [15] and psychiatric disorders [16,17], such as depression, anxiety, and conduct disorders. The societal costs of untreated perinatal mood disorders for all US births in 2017 were estimated at US $14.2 billion [18], and unfortunately, half of the women with a perinatal mood disorder (which includes PPD) do not receive the treatment they need.

Recent guidelines by the United States Preventive Services Task Force [19] and several specialized medical societies, including the American Academy of Pediatrics and the American College of Obstetricians and Gynecologists [20,21], have established perinatal depression screening and treatment as essential components of postpartum care. Despite these recommendations, several patient- and system-level barriers to the receipt of current treatment options (eg, psychotherapy and antidepressant medications) remain. For example, although psychotherapy is an effective nonpharmacological treatment option, numerous barriers to receiving care exist, including the shortage of mental health care providers [22], limited access to care, financial constraints, and lack of time, transportation, and childcare [23]. In addition, most pregnant and postpartum women (83-95%) prefer nonpharmaceutical treatments [24]. Further, the COVID-19 pandemic has had a significant impact on the mental health of pregnant and postpartum women, with more than one-third of women reporting significant depression symptoms [25]. Although the rates of perinatal depression have increased, access to in-person delivered health care has drastically diminished [26,27]. Therefore, it is critical to identify safe, effective, patient-centered, and scalable intervention options for postpartum women with heavy depression symptom burden.

### Mindfulness Interventions for Depression

Mindfulness, a psychological process of bringing attention to the present moment [28-30], has demonstrated its effectiveness as an intervention for reducing symptoms of depression in many populations [31-35]. However, gold standard mindfulness training often requires 30 or more hours of *in-person* instruction with 45 min of daily homework [28,36]. Thus, despite their known efficacy in reducing depression symptoms, traditional mindfulness programs pose similar accessibility barriers to those associated with counseling services, reducing its potential to help busy women with PPD. Technology is becoming an increasingly popular method for delivering lifestyle and behavioral interventions, and there has been a steady rise in mobile health (mHealth) interventions, particularly as they fit the lifestyles of individuals who are unable to attend regular in-person classes. Thus, a self-paced, mHealth mindfulness-based intervention has potential as a scalable behavioral intervention that addresses barriers to traditional mindfulness programs.

Research is needed to ascertain the effectiveness of mHealth mindfulness interventions in postpartum women with depressive symptoms. As a first step, we conducted a feasibility study of an mHealth mindfulness-based intervention for women with moderate to moderately severe PPD symptoms within a large integrated health care delivery system. This study investigates the feasibility and acceptability of the mHealth mindfulness intervention while also reporting on the preliminary efficacy of patient-reported outcomes to determine whether conducting a randomized control trial of the intervention is warranted.

## Methods

### Study Setting

The study was conducted within Kaiser Permanente Northern California (KPNC), an integrated health care delivery system serving over 4.4 million racially and socioeconomically diverse members representative of the Northern California population [37,38]. Standard postpartum care includes screening for depression at the fourth to eighth week postpartum visit using the 9-item Patient Health Questionnaire (PHQ-9) [39,40].

### Study Design and Population

A mixed-methods single-arm trial of a 6-week mHealth mindfulness intervention was conducted between March 2018 and June 2019. Women seeking postpartum care were recruited from 7 of the 44 KPNC obstetrics and gynecology clinics. Women aged at least 18 years, within 6 months of giving birth, with a PHQ-9 score of 10 to 19 (indicating moderate to moderately severe depressive symptoms), English-speaking, with access to a smartphone, tablet, or computer with internet access were eligible for the study. Women who engaged in regular mindfulness, meditation, or yoga practice 3 or more times per week or enrolled in a mindfulness program were excluded. Participants were asked to complete a web survey at baseline and immediately after the intervention to assess patient-reported outcomes of depression, stress, sleep quality, and mindfulness. Semistructured interviews were conducted within 3 weeks of completion of the intervention to assess the acceptability of the mHealth mindfulness intervention. This study was approved by the KPNC institutional review board.

### Participant Identification and Recruitment

Potential participants were identified via 2 strategies: (1) a postpartum PHQ-9 score of 10 to 19, identified through the KPNC electronic health records (EHRs), and (2) self- or clinician-referral from KPNC obstetrics and gynecology clinical staff or study brochures. Potential eligible participants were contacted about the study via email and phone by a research assistant and rescreened for depression symptoms using the 8-item Patient Health Questionnaire (PHQ-8; see the *Measures* subsection for more information). Women who met all eligibility criteria and had a PHQ-8 score of 10 to 19 were enrolled. Participants who completed both baseline and 6-week follow-up surveys received a US $25 gift card and an additional year-long subscription to the mindfulness app.

### Intervention

On signing the informed consent and completing the baseline survey, participants were provided access to a commercially available mindfulness app, Headspace. Headspace was chosen because it was identified as the best commercially available mindfulness mobile app in a review published in a peer-reviewed journal [41], and most of the previous studies, including ours, have reported that Headspace is an accessible and effective tool for delivering training to increase mindfulness in various populations [42-48]. Headspace provides self-paced, guided mindfulness meditations through a website or mobile app (iOS and Android). The home screen displays the next meditation in the series. Much of the program follows a linear pathway of daily, progressive meditations (ie, each day builds upon previous content) designed to deepen the understanding of mindfulness and encourage its integration into daily life.

The women were asked to use the app for 10 to 20 min a day during the 6-week study period. Each participant was given a study-specific log-in ID and encouraged to complete the 30-day *Basics* course first and then choose from the other themed sessions (eg, anxiety, relationships) for the remainder of the 6-week study period. When the study staff noted that a participant had completed fewer than 3 sessions in the past week, they called the participant to remind her to use the app.

### Measures

#### Feasibility

We assessed 2 feasibility measures: adherence and retention.

- Retention: retention was calculated as the proportion of enrolled participants who completed both the baseline and postintervention surveys;
- Adherence to the intervention: the date, time, duration, and type of each meditation session that participants completed were collected by Headspace using the study-specific log-in ID. Adherence was assessed for all enrolled women and for women who completed the study.

#### Acceptability

Acceptability was assessed through responses in a semistructured interview. Participants were asked open-ended questions about their experience with the study and Headspace, recommended changes to the study procedures, perceived effects or benefits of practicing mindfulness, and perceived need for additional health system support for pregnant and postpartum women. Participants were also asked to respond to the question, “What was your overall experience with the Headspace program?” with 1 of the 4 responses: extremely useful/satisfied, very useful/satisfied, somewhat useful/satisfied, and not at all useful/satisfied. Participants' responses were written down as close to verbatim as possible by the interviewer.

#### Preliminary Efficacy of Participant-Reported Outcomes

The 4 participant-reported outcomes assessed were depression, stress, sleep quality, and mindfulness.

- Depression: the PHQ-8 [49] depression screener is a validated instrument adapted from the PHQ-9, which was used to assess current depression symptoms at recruitment and follow-up. The PHQ-8 excludes the question regarding suicidal thoughts. The PHQ-8 scores ranged from 0 to 24. Scores of 1 to 4 suggest minimal depression, 5 to 9 mild depression, 10 to 14 moderate depression, 15 to 19 moderately severe depression, and 20 to 24 severe depression;
- Stress: the 10-item Perceived Stress Scale [50] assesses the degree to which a respondent perceives situations in his or her life in the previous month as stressful through a 5-point Likert scale (0=*never* to 4=*very often*). The scores are summed to give a total score ranging from 0 to 40;
- Sleep quality: the 19-item Pittsburgh Sleep Quality Index [51] asks about sleep quality during the previous month, including questions on sleep duration, sleep disturbance, and use of sleep-inducing medications. A global score ranging from 0 to 21 is calculated using 7 components of sleep. Higher scores indicate poorer sleep quality;
- Mindfulness: the 24-item Five Facet Mindfulness Questionnaire-Short Form [52] uses a 5-point Likert scale to measure mindfulness and includes subscales to assess 5 elements of mindfulness—observing, describing, acting with awareness, nonjudging of inner experience, and nonreactivity to inner experience. Responses vary between 1 (never or very rarely true) to 5 (very often or always true). Scores for overall mindfulness range from 24 to 120. The scores for observing range from 4 to 20, whereas the scores for describing, acting with awareness, nonjudging of inner experience, and nonreactivity to inner experience range from 5 to 25. Higher scores indicate greater mindfulness.

### Analytic Methods

#### Quantitative Data Analyses

Baseline characteristics differences between completers and noncompleters were assessed using the analysis of variance (ANOVA) for continuous variables and the Fisher exact test for categorical variables. Baseline mean and SD were calculated for all participant-reported outcome measures. Pre-post changes in scores and *P* values were derived using paired *t* tests, and a repeated measures ANOVA analysis was conducted to compare pre-post changes in scores by adherence to the intervention (meditated <50% of the days vs ≥ 50% of the days). All analyses were conducted using SAS software version 9.4 (Cary, NC).

#### Qualitative Assessment and Analysis

Qualitative data were uploaded into the NVivo qualitative data analysis software (QSR International Pty Ltd version 12, 2018). Inductive thematic analysis was used to identify and develop codes on themes related to mindfulness benefits, interface experience, experience with the study, and suggested changes. Each interview was coded by 2 primary coders (MM and LN); a third coder (EK) reviewed all coded transcripts to ensure the accuracy of codes.

## Results

### Recruitment

**Figure 1:**
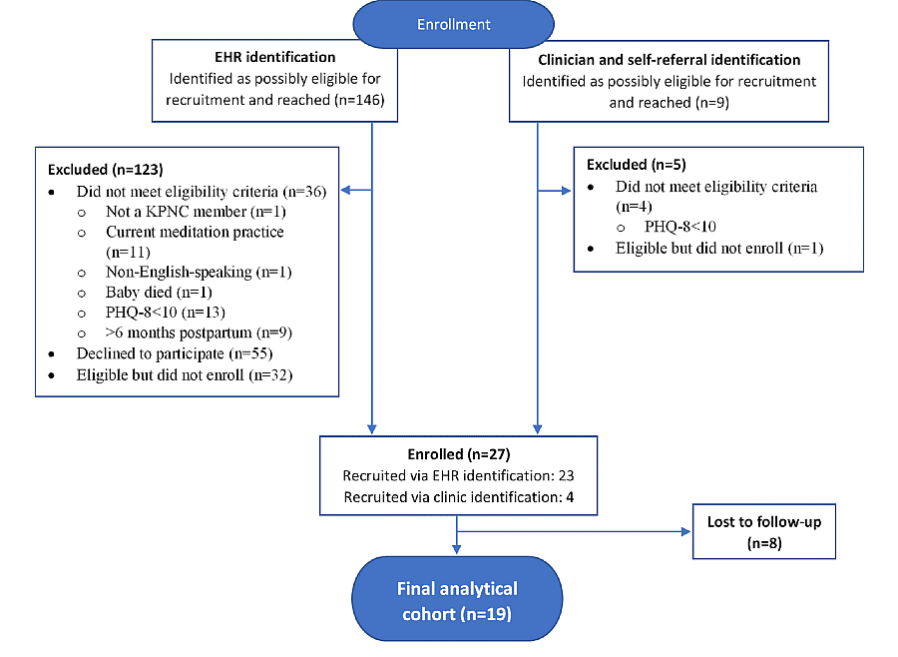
Study recruitment flowchart for a mobile health mindfulness feasibility study for postpartum women with moderate to moderately severe symptoms of depression in Kaiser Permanente Northern California between 2018 and 2019.

We contacted and reached 155 potentially eligible women, identified through the EHR (n=146) or clinician referral (n=9), by phone and assessed their eligibility; some women declined to participate (Figure 1). Of the women who were contacted, 26% (40/155) did not meet the eligibility criteria, 36% (55/155) declined to participate, and 21% (33/155) were eligible and interested but did not complete the enrollment process and thus were excluded from the study. The most common reasons for ineligibility were PHQ-8 scores outside of the eligible range and existing meditation practice. The most common reasons for declining participation were being too busy, too tired, already sought other treatments for depression, and lack of interest. A total of 27 of the 115 women who were either eligible or who declined to participate before eligibility was assessed, enrolled in the study, corresponding to a conservative 24% (27/115) recruitment rate.

### Baseline Characteristics

Of the 27 women recruited, 9 (33%) were Hispanic, 8 (30%) were White, and 5 (19%) were Black. More than half (15/27, 56%) of the participants did not have a college degree, nearly a quarter (6/27, 22%) received Medicaid benefits, and most (20/27, 74%) had household income <US $100,000. The mean baseline PHQ-8 score was 14.3 (range 10-23, SD 3.8), and about half of the women had a current depression diagnosis (13/27, 48%; 6/27, 22%; postpartum only; and 7/27, 26%, postpartum and history of depression; Table 1). There were no significant differences between women who completed the study and those who did not complete it.

**Table 1:** Baseline characteristics of participants in a feasibility mobile health mindfulness study of postpartum women with moderate to moderately severe symptoms of depression in Kaiser Permanente Northern California between 2018 to 2019, overall and by study completion status.

Characteristics		Total (n=27)	Completed (n=19)	Not completed (n=8)
Baseline PHQ-8^a^ score, mean (SD)		14.3 (3.8)	15.2 (4.0)	12.1 (2.0)
Number of weeks postpartum, mean (SD)		12.3 (5.7)	11.5 (5.0)	14.3 (7.2)
Age (years), mean (SD)		30.9 (5.2)	31.4 (5.3)	29.6 (5.1)
**Depression diagnosis, n (%)**				
	Postpartum only	6 (22)	4 (21)	2 (25)
	History of depression and postpartum	7 (26)	7 (37)	0 (0)
	None	14 (52)	8 (42)	6 (75)
**Race and ethnicity, n (%)**				
	Non-Hispanic White	8 (30)	7 (37)	1 (13)
	Non-Hispanic Black	5 (19)	3 (16)	2 (25)
	Asian	1 (4)	1 (5)	0 (0)
	Hispanic	9 (33)	4 (21)	5 (63)
	Multiracial	4 (15)	4 (21)	0 (0)
**Education, n (%)**				
	Less than college graduate	15 (56)	9 (47)	6 (75)
	College graduate	12 (44)	10 (53)	2 (25)
**Household income, n (%)**				
	<US $100,000	20 (74)	13 (68)	7 (88)
	≥US $100,000	7 (26)	6 (32)	1 (13)
**Medicaid status, n (%)**				
	Yes	6 (22)	2 (11)	4 (50)
	No	18 (67)	15 (79)	3 (38)
	Unknown	3 (11)	2 (11)	1 (13)
**Marital status, n (%)**				
	Married/living with partner	19 (70)	15 (79)	4 (50)
	Single	7 (26)	3 (16)	4 (50)
	Unknown	1 (4)	1 (5)	0 (0)
**Parity, n (%)**				
	0	13 (48)	10 (53)	3 (38)
	1+	14 (52)	9 (47)	5 (63)
**Primary device used for the mindfulness program, n (%)**				
	Android	5 (19)	5 (26)	0 (0)
	iOS^b^	15 (56)	14 (74)	1 (13)
	Nonparticipator	7 (26)	0 (0)	7 (88)

^a^PHQ-8: 8-item Patient Health Questionnaire.

^b^iOS: iPhone OS.

### Feasibility Outcomes

#### Retention

Seventy percent (19/27) of participants completed the baseline and follow-up surveys (Table 1).

#### Adherence to the Intervention

High rates of engagement were noted among the participants. Of the 27 women who enrolled in the study, 20 (74%) used the Headspace app at least once, with 9 (33%) practicing meditation for at least half of the days during the 6-week study period and 5 (19%) practicing at least 70% of the days. These rates were similar when only women who completed the study were considered. Among the women who completed the study, 100% (19/19) used the Headspace app at least once. Of these 19 participants, 9 (47%) practiced meditation for at least half of the days during the 6-week study period, and 5 (26%) practiced meditation for at least 70% of the days. In addition, 58% (11/19) participants used Headspace at least once during the month after the 6-week intervention period ended.

#### Acceptability

Of the participants who completed the study, 84% (16/19) participated in a postintervention telephone interview; 11 participants (11/16, 69%) responded that they were either *very* or *extremely* satisfied with the Headspace app, and all of the participants planned to continue their mindfulness practice with Headspace after the study ended. Results from the semistructured interviews supported participant satisfaction, as women reported that they felt it was easy to use, liked the variety of available meditation options, and valued the convenience of an app-based intervention. Whereas some participants liked the male facilitator's voice for meditation, others shared that they would prefer different voice options to guide the meditations, particularly a female voice, an option that Headspace added during the course of the study. Two participants wanted more freedom to explore different meditation sessions rather than to follow the prescribed course required by the study; 1 had meditation experience and would have liked to start at a more advanced level than to follow the basic meditation course; the other was interested in trying shorter (eg, 1 min) meditations because she found it hard to remain uninterrupted with a newborn. Another participant would have liked more phone calls from study staff to assist with initially downloading the app and weekly check-ins to encourage the use of the app.

Participants noted several benefits of using Headspace. The most commonly mentioned benefits were improved stress management, reduced anxiety, improved sleep, and increased physical activity. Many also noted that meditation allowed them to take some time off for themselves. Several participants liked having a structured routine of meditating each day. Others said they would work meditation, particularly the counted breathing technique, into their day while driving, exercising, or in moments of stress (eg, when the baby was crying).

### Preliminary Efficacy of Participant-Reported Outcomes

**Figure 2:**
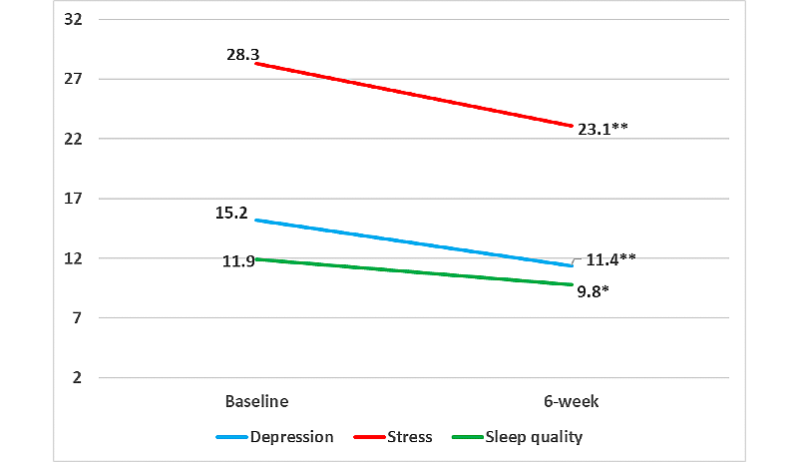
Differences in pre- and postintervention patient-reported outcomes in a mobile health mindfulness feasibility study of postpartum women with moderate to moderately severe symptoms of depression in Kaiser Permanente Northern California between 2018 and 2019. *P<.05 and **P<.01.

**Figure 3:**
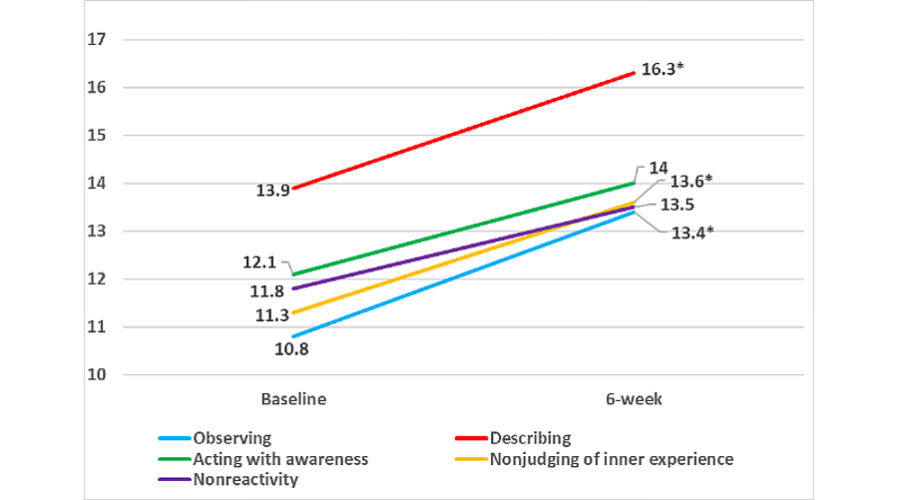
Differences in pre- and postintervention patient-reported mindfulness outcomes in a mobile health mindfulness feasibility study of postpartum women with moderate to moderately severe symptoms of depression in Kaiser Permanente Northern California between 2018 and 2019. *P<.05 and **P<.01.

**Figure 4:**
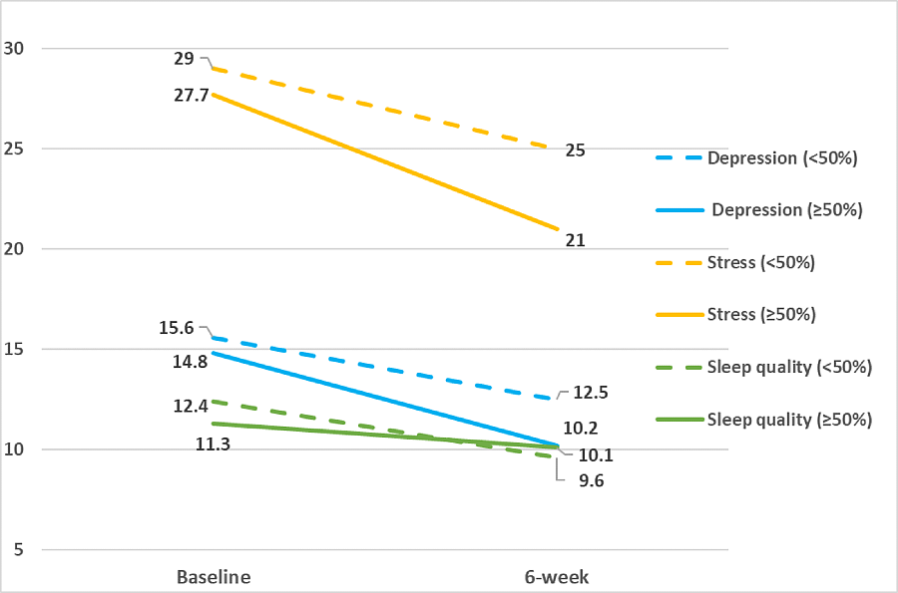
Patient-reported outcomes by percent Headspace intervention adherence in a mobile health mindfulness feasibility study of postpartum women with moderate to moderately severe symptoms of depression in Kaiser Permanente Northern California between 2018 and 2019.

At the 6-week postintervention follow-up assessment, participants experienced significant improvements in depressive symptoms (−3.8, SD 5.0, *P*=.004), perceived stress (−6.0, SD 7.9, *P*=.005), and sleep quality (−2.1, SD 3.4, *P*=.02, indicating less sleep disturbance) compared with baseline (Figure 2). Participants also achieved significantly greater levels of mindfulness in 3 of the 5 mindfulness domains (observing: 2.5, SD 3.9, *P*=.01; describing 2.4, SD 4.2, *P*=.02; nonjudging of inner experience 2.4, SD 4.1, *P*=.02; Figure 3) and significant improvements in overall mindfulness (10.9, SD 16.8, *P*=.01). Although not statistically significant, trends suggest a greater improvement in depression symptoms (−4.6, SD 5.2 vs −3.1, SD 4.9, *P*=.54), stress (−6.7, SD 8.8 vs −5.3, SD 7.3, *P*=.55; Figure 4), and overall mindfulness (13.3, SD 18.4 vs 8.8, SD 15.8, *P*=.57) for women who meditated using the app for at least 50% of the days compared with women who meditated for less than 50% of the days of the 6-week intervention, respectively. No differences were noted in sleep quality between the groups (−1.2, SD 3.9 vs −2.8, SD 2.8, *P*=.32; Figure 4).

## Discussion

### Principal Findings

Findings from this study suggest that a self-paced mHealth mindfulness intervention for women with moderate to moderately severe symptoms of PPD is both feasible and acceptable. This study demonstrated our team's ability to identify and recruit postpartum women with significant depression symptoms with good recruitment and retention rates. The participants appreciated the convenience of the intervention: most of the women engaged in the meditation program at least one time and a third of all recruited participants and nearly half of those who completed the study meditated for a majority of the intervention days. The quantitative data and findings from the semistructured interviews suggest preliminary efficacy and improvement in depression symptoms, stress, sleep, and mindfulness in postpartum women with moderate to moderately severe symptoms of PPD, suggesting that a full-powered trial is warranted.

This feasibility study also provided information on study protocols that may be used to improve the efficiency of a larger efficacy trial. Tracking participants' app usage allowed us to reach out when there was an extended period of inactivity (ie, <3 sessions in the previous week). This helped remind some participants to get back on track with the app. Using the reminder features and push notifications built into the app may also be useful as a reminder, given that at least 1 participant shared that they would have liked weekly reminders from the study team to encourage the use of the app. Further, automatic tracking of progress can be used as a source of motivation to continue using the app. Such interactive features of a mobile app can increase its adherence and effectiveness, and ease of use can facilitate widespread, efficient implementation [53]. Additionally, the assessment of patient-reported outcomes provides preliminary effect estimates for informing power in a randomized control trial.

### Comparison With Previous Work

The theoretical basis for mindfulness derives from a model based on studies of the influence of mindfulness on brain regions involved in learning and memory processes, emotion regulation, self-referential processing, and perspective taking [54,55]. Recent research suggests that mindfulness programs may serve as a nonpharmacological treatment option for postpartum women with depression. For example, a recent meta-analysis, which included 9 studies of adults with depression (75% women), documented a significantly reduced risk of relapse over a 5-year follow-up period for those who received a mindfulness-based intervention compared with those who did not (hazard ratio, 0.69; 95% CI 0.58-0.82) [56]. Additionally, a meta-analysis of 8 randomized controlled trials of pregnant women concluded that women in the in-person mindfulness arm experienced significant reductions in depression [34].

Despite the positive effects of mindfulness programs on depression symptoms, the requirements of traditional mindfulness programs limit the accessibility and adherence of postpartum women. Traditional mindfulness programs often require more than 30 hours of *in-person* instruction along with 45 min of home practice daily. Postpartum women often face competing priorities such as a child at home or a full-time job, making attendance at in-person sessions challenging. A recent in-person mindfulness pilot study for pregnant women reported that recruitment was challenging for these reasons and that a more practical intervention was needed [32]. These findings may also be generalized to postpartum women. A recent meta-analysis including 65 randomized controlled trials, totaling 5489 participants, demonstrated that brief mindfulness training (ranging from a single-session to multisession interventions lasting up to 2 weeks) was also effective in reducing depression and anxiety [57]. Given the busy lifestyle of new mothers, convenient, frequent, and low dosage mindfulness programs that can be accessed from anywhere are more feasible than the traditional, lengthy, and in-person mindfulness interventions.

In addition to these common barriers, the COVID-19 pandemic has caused a surge in PPD symptoms, increasing the demand for mental health care. A recent study documented a near tripling of perinatal depression, with 15% of women reporting high depression symptoms prepandemic compared with 41% reporting such symptoms since the pandemic started [58]. Our study was conducted before COVID-19, and reasons for women declining participation included already seeking other treatments for depression and being too busy, although being too busy was a barrier this intervention was developed to address. The sheltering-in-place orders have had a dramatic impact on busyness, whereas the COVID-19 pandemic has simultaneously had a significant impact on mental health care resources, decreasing the availability of other options for depression treatment. Further, it is not clear whether the women felt that participating in an intervention study would be time consuming or the actual intervention would be so. Thus, as perinatal depression has increased during this time, the lifestyles of people have changed, and access to in-person delivered health care has greatly decreased [26,27], further highlighting the need for effective, remotely delivered interventions.

Recent studies report that many Americans having depression, stress, or anxiety prefer internet-based mindfulness training over in-person sessions [59,60]. A recent meta-analysis of web-based, webinar-versions of traditional mindfulness-based interventions (eg, Mindfulness-based Stress Reduction) demonstrated a significant beneficial impact on stress, anxiety, depression, and well-being [61]. Although the use of technology for the delivery of mindfulness programs is a major advancement, these studies used web-based versions of the traditional mindfulness interventions in a webinar format with a facilitator, which still required the participants to log on weekly at a specified time, and had the same extensive training duration (eg, 30 or more hours, 2.5 hours per session) and homework requirements (45 min per day) as the in-person versions [62-65]. Although the use of technology can help increase accessibility to the intervention compared with in-person sessions, this type of intervention can still be resource-intensive, and thus not readily scalable, and be still challenging for new mothers who often do not have flexible schedules to attend scheduled sessions. Our study addressed these barriers by offering a web-based, self-paced, and brief mindfulness intervention.

Women in our study valued the variety of options provided by the mindfulness program. They enjoyed both the guided meditations and the breathing techniques and the ability to choose from a male or female voice. Additionally, women also reported the perceived benefits of the program, such as improved stress management, reduced anxiety, improved sleep, and increased physical activity and techniques for managing stress in stressful moments (eg, baby crying). Most of the women contacted for potential recruitment into the study had access to the technology required, and very few participants had trouble downloading or using the app. Although our study was not able to assess an effective daily dose, future studies should consider assessing the effectiveness of various dosages (duration and frequency). However, overall, women enjoyed the intervention.

Our results also suggest the potential for mHealth mindfulness-based interventions to reach women of low socioeconomic status, a population that often does not have access to more traditional treatment options (eg, psychotherapy) for PPD. Nearly a quarter of our sample received Medicaid benefits during the postpartum period. Women of low socioeconomic status are at increased risk of depression, and most women with a depression diagnosis do not receive the treatment they need [66]. Mobile devices are becoming increasingly popular, and approximately 90% of Americans of reproductive age own a smartphone [67]. This type of intervention will also be cost saving for health care systems that have limited resources to offer mental health services, particularly during the COVID-19 pandemic where health care resources are scarce, and distress rates are high.

### Limitations

This feasibility study has several limitations. First, we are not able to draw conclusions regarding the efficacy of the mHealth mindfulness intervention on participant-reported outcomes or assess dose-response relationships given the small, single-arm feasibility study design. Without a control group, it is not possible to know whether the observed improvements can be attributed to the intervention. However, we successfully demonstrated the feasibility and acceptability of the intervention, which the study was designed to evaluate. Further, the preliminary efficacy results on patient-reported outcomes assessed immediately after the intervention were promising; however, we acknowledge that these results could be affected by missing data. The positive feasibility, acceptability, and preliminary efficacy findings support the need for a future, larger effectiveness study with longer-term follow-up. Second, the generalizability may be limited because we required the use of technology as part of the intervention. However, as described above, most women who we attempted to recruit owned mobile devices or computers with an internet connection, making the *digital divide* unlikely. The main reasons reported for declining to participate were lack of time or interest and not lack of access to technology. Third, the intervention was only offered in English, and thus non-English speakers were ineligible. Future mHealth mindfulness-based studies should incorporate other languages, given the high burden of depression among minority populations [68]. Of note, Headspace now offers programs in other languages, offering opportunities to conduct more generalizable studies.

### Conclusions

Findings from this study demonstrate that postpartum women with moderate to moderately severe depressive symptoms are interested in a mobile-based mindfulness intervention. In addition, we demonstrate that conducting an mHealth mindfulness intervention study in this population is feasible within a large integrated health care system. Larger-scale randomized trials are needed to establish the efficacy and effectiveness of mHealth mindfulness interventions in this population. Highlighting the need for such studies is the recent recommendation by the United States Preventive Services Task Force to refer all women at increased risk of perinatal depression to counseling services. Implementing this recommendation will place a tremendous burden on the health care system, which already has a shortage of mental health care providers [69-74]. Thus, the findings from this study support the need for pragmatic trials, which will provide evidence on the effectiveness of implementing low-cost, technology-based programs for women with moderate to moderately severe PPD symptoms, which are necessary for improving the health of families.

## Abbreviations

ANOVA: analysis of variance
EHR: electronic health record
KPNC: Kaiser Permanente Northern California
mHealth: mobile health
PHQ-8: 8-item Patient Health Questionnaire
PHQ-9: 9-item Patient Health Questionnaire
PPD: postpartum depression
